# P36 Awareness and uptake of the AMS/AMR indicative curriculum for undergraduate student pharmacists in UK schools of pharmacy

**DOI:** 10.1093/jacamr/dlag102.042

**Published:** 2026-06-26

**Authors:** Naomi Fleming, Antonella Tonna

**Affiliations:** School of Pharmacy, Applied Sciences and Public Health, Robert Gordon University, Aberdeen, Scotland; School of Pharmacy, Applied Sciences and Public Health, Robert Gordon University, Aberdeen, Scotland

## Abstract

**Background:**

In 2022, a National Antimicrobial Stewardship Pharmacy Education group (NAPEG) was formed comprising academics from Schools of Pharmacy (SOP), AMS specialist pharmacists, NHS England, NHS Wales, NHS Scotland, UK Health Security Agency, British Society of Antimicrobial Chemotherapy, British Pharmacy Students Association, Royal Pharmaceutical Society and UK Clinical Pharmacy Association. The group co-designed the undergraduate pharmacy AMR/AMS indicative curriculum with NHSE (published February 2025), along with a supporting competency-based framework and practice-based assessment framework for designated supervisors. The group worked with BSAC to identify and develop content for the pharmacy section of Keep Antibiotics Working.

**Objectives:**

To determine the awareness and uptake of the AMR/AMS indicative curriculum and associated resources for undergraduate student pharmacists within SOP, one year since publication.

**Methods:**

A Teams survey was sent to all 36 NAPEG SOP members to ascertain awareness of the different resources and their intentions to use them or actions to date. This was circulated between October 2025 and February 2026 through SOP representatives on NAPEG.

**Results:**

There were 25 responses out of 36 SOP (7 SOP will recruit the first cohorts in September 2026). Of these 25, 10 respondents had mapped the indicative curriculum for AMR/AMS to their school curriculum; 7 had used it to find content, while 10 intended to use it. Only one respondent was not aware of the curriculum. Seven respondents had used the AMR/AMS competency framework to map to their school curriculum. Seven has used it to find content and ten intended to use it. Three respondents were not aware of it. When asked about the AMR/AMS practice-based framework for designated supervisors, 3 respondents had used this to map to their school curriculum, four had used it to find content, seven intended to use it. Three respondents were not aware of it. Ten respondents had used the BSAC KAW pharmacy resource to find content for their AMR/AMS teaching, but 3 respondents were not aware of it. Manifest content analysis of free text responses showed the following key themes: Respondents indicated using the AMR/AMS curriculum and frameworks and the BSAC KAW resources to generate ideas and identify content describing the resources as ‘very helpful.’ An application beyond pharmacy was also described where resources were seen ‘to inform interprofessional education.’ Newer SOP had not implemented the curriculum but intended to use it and the resources for future programme development. Despite reporting an awareness of the practice-based framework, some reported barriers to using it due to ‘too many mapping documents’ and concerns about ‘introducing too many frameworks’ for student pharmacists and trainees.

**Conclusions:**

Most respondents are aware of the resources and 40% had mapped their school curricula content to the national indicative curriculum. Despite co-design of the curricula and associated resources with the NAPEG group, implementation in individual SOP is still inconsistent a year on. This may reflect the fact that the curriculum is *indicative* at this stage and therefore there is no requirement for SOP to implement this reflecting a need for *mandating* the curriculum.

